# Update on Cerebellar Ataxia with Neuropathy and Bilateral Vestibular Areflexia Syndrome (CANVAS)

**DOI:** 10.1007/s12311-020-01192-w

**Published:** 2020-10-04

**Authors:** Mathieu Dupré, Ruben Hermann, Caroline Froment Tilikete

**Affiliations:** 1 Neuro-ophthalmology Unit, Hopital Neurologique et Neurochirurgical P Wertheimer, Hospices Civils de Lyon, Lyon, France; 2 ENT, Cervico-Facial Surgery and Audiophonology, Hôpital Edouard Herriot, Hospices Civils de Lyon, F-69003 Lyon, France; 3 Lyon Neuroscience Research Center, IMPACT Team, INSERM, U1028, CNRS, UMR5292, Lyon, France; 4 Lyon I University, F-69373 Lyon, France

**Keywords:** Neuronopathy, Ganglionopathy, Bilateral vestibulopathy, Head impulse test, Visuo-vestibulo-ocular reflex, RFC1

## Abstract

The syndrome of cerebellar ataxia with neuropathy and bilateral vestibular areflexia (CANVAS) has emerged progressively during the last 30 years. It was first outlined by the neurootology/neurophysiology community in the vestibular areflexic patients, through the description of patients slowly developing late-onset cerebellar ataxia and bilateral vestibulopathy. The characteristic deficit of visuo-vestibulo-ocular reflex (VVOR) due to the impaired slow stabilizing eye movements was put forward and a specific disease subtending this syndrome was suggested. The association to a peripheral sensory axonal neuropathy was described later on, with neuropathological studies demonstrating that both sensory neuropathy and vestibular areflexia were diffuse ganglionopathy. Clinical and electrophysiological criteria of CANVAS were then proposed in 2016. Besides the classical triad, frequent chronic cough, signs of dysautonomia and neurogenic pains were frequently observed. From the beginning of published cohorts, sporadic as well as familial cases were reported, the last suggestive of an autosomal recessive mode of transmission. The genetic disorder was discovered in 2019, under the form of abnormal biallelic expansion in the replication factor C subunit 1 (RFC1) in a population of late-onset ataxia. This pathological expansion was found in 100% of the familial form and 92% of sporadic ones when the triad was complete. But using the genetic criteria, the phenotype of CANVAS seems to expand, for exemple including patients with isolated neuronopathy. We propose here to review the clinical, electrophysiological, anatomical, genetic aspect of CANVAS in light of the recent discovery of the genetic aetiology, and discuss differential diagnosis, neuropathology and physiopathology.

**Electronic supplementary material:**

The online version of this article (10.1007/s12311-020-01192-w) contains supplementary material, which is available to authorized users.

## Introduction

The association of vestibular dysfunction to chronic cerebellar ataxia has for long time been reported in patients with hereditary cerebellar ataxia, such as spinocerebellar ataxia type 3 (SCA3) [1], SCA1 [2], Friedreich’s ataxia [3] or primary neurodegenerative diseases considered as olivo-ponto-cerebellar (OPCA) form of multisystem atrophy (MSA) [4].

In the 1990s, neurootologists began to identify patients with a pattern of slowly progressive vestibular dysfunction and cerebellar ataxia that did not correspond to known etiologies. Their interest was first neurophysiological in showing absence of compensatory cervico-ocular reflex [5] and of compensatory smooth pursuit [6] during visuo-vestibular ocular reflex (VVOR) in 2 patients for each study. The quite frequent association of cerebellar ataxia to bilateral vestibulopathy was then reported in a retrospective series of 53 patients [7]. Seven (13%) of these patients presented with cerebellar degenerative ataxia, 1 with confirmed Friedreich’s ataxia, 1 with probable SCA (autosomal dominant heredity) and 1 with consanguine family. Peripheral sensory neuropathy was reported in 4 out of the 7 patients. The authors suggested that vestibular failure may be underdiagnosed in patients with cerebellar degenerations.

The question of a distinct syndrome was raised in 2004, with the clinical and electrophysiological description of a series of 4 patients, with a quite specific pattern of slowly progressive adult-onset cerebellar ataxia with bilateral vestibulopathy [8]. In these patients in whom SCA 1, 2, 3, 6, 7 and Friedreich’s ataxia were excluded by genetic testing, the objective was still to analyse and validate its characteristic clinical sign: impairment of the VVOR. VOR was also profoundly affected, as for idiopathic vestibular areflexia. Among these four patients, 3 had clinical and electrophysiological evidence of a sensory peripheral neuropathy; one of them with severe axonal neuropathy on sural nerve biopsy. The authors suggested that “the clinical pattern does not fit any single presently accepted nosologic entity”. Indeed, none of the patient had acquired cause of cerebellar ataxia; the depth of vestibular deficit was different from what is observed in SCA3, SCA1 and Friedreich’s ataxia, and not associated to deafness such as in Friedreich’s ataxia; the slow progression of the disease, the absence of extrapyramidal signs and autonomic disorders did not fulfil the currently clinical criteria for MSA. They suggested a novel syndrome that was called *cerebellar ataxia with bilateral vestibulopathy*, but still questioned the pure coincidence of the association. The authors did not include the peripheral neuropathy within the syndrome.

The non-coincidental association of cerebellar ataxia, bilateral vestibulopathy *and peripheral neuropathy* was confirmed in 2007, by two studies from the same team. First, in a retrospective cohort of 255 patients with bilateral vestibulopathy, Zingler et al. [9] demonstrated that 25% presented with cerebellar ataxia, and among them 32% had neuropathy. Cerebellar ataxia was slowly progressive in the majority of patients, and the neuropathy was described as predominantly sensory and axonal. The authors suggested a novel syndrome that may be due to a neurodegenerative or autoimmune process. It is also noticeable there that 18% of patients with bilateral vestibulopathy without cerebellar ataxia presented with a peripheral neuropathy. Second, in a retrospective cohort of 117 patients with downbeat nystagmus, Wagner et al. [10] found 45 patients (38%) without unidentifiable cause of DBN, called “idiopathic DBN”, in whom 16 were associated with at least two of the following: bilateral vestibulopathy, cerebellar signs and peripheral neuropathy. The peripheral neuropathies were heterogeneous, including sensory, motor, demyelinating, axonal and mixed types. Cerebral MRI was found normal in these patients. The authors suggested that “the association of these disorders suggest that this syndrome may reflect a multisystem neurodegeneration or channelopathy”.

Vestibular deficit in polyneuropathy is quite frequent and has notably been demonstrated for example in chronic inflammatory demyelinating polyneuropathy [11] and later in demyelinating polyneuropathy [12], in whom 2/3 of patients showed unilateral (approximately 50%) or bilateral (approximately 50%) gain reductions of the horizontal high-acceleration VOR. However, in these studies, none of the patients presented with cerebellar ataxia and the vestibular deficit was not deep. The authors mainly suggested a common pathomechanism of peripheral nerve and vestibular nerve involvement.

In 2011, the specific entity associating late-onset slowly progressive cerebellar ataxia, bilateral vestibular areflexia and axonal sensory neuropathy was fully described in a cohort of 18 patients and renamed *cerebellar ataxia with neuropathy and bilateral vestibular areflexia syndrome* (*CANVAS*) [13]. The same team later on published a retrospective cohort of 27 patients, with a mean age at symptom onset of 60 years and patients seen with a mean of 11 years after symptom onset [14]. Vestibular testing showed absent or severely reduced vestibulo-ocular reflex on different frequencies. Cerebellar ataxia was not specific, and associated to cerebellar atrophy on MRI. They showed systematic non-length-dependent sensory deficit with absent sensory nerve action potentials. Temporal bone histopathology in one case showed neuronopathy (ganglionopathy) of the vestibular nerve, facial and trigeminal nerve and preservation of the auditory nerve [15]. This suggested that the peripheral neuropathy could be a neuronopathy [16]. The involvement of cerebellum or peripheral nerve could be subtle in some patients. The progressions were variable, but mainly slow, with no handicap for decades. Since 2 pairs of siblings were observed in their cohort, the authors suggested a late-onset recessive disorder. In addition to the triad, the same team showed in a cohort of 80 patients, frequent chronic cough, orthostatic hypotension and neuropathic pain [17]. The autonomic disorder was also described by other teams [18].

Even though, proposed diagnostic criteria in 2016 of definite CANVAS were abnormal VVOR on videooculography, videonystagmography or rotational chair testing, and cerebellar atrophy on MRI displaying anterior and dorsal vermis atrophy and lateral hemispheric atrophy predominantly affecting crus I, and neurophysiologic evidence of a neuronopathy (ganglionopathy), and exclusion of genetic ataxias able to be gene tested, particularly SCA3 and Friedreich ataxia [19].

Spasmodic cough was further shown to be an integral part of the clinical picture in CANVAS, antedating the appearance of imbalance in several decades [20] as well as normal tendon reflex while the patients were suffering neuronopathy [21].

In 2019, Cortese et al. identified a recessive repeat expansion in intron 2 of RFC1 as the main cause of CANVAS [22]. They indeed found this recessive expansion in 100% of familial CANVAS (*n* = 23) and 92% of sporadic CANVAS. Furthermore, out of 150 cases diagnosed with familial or sporadic late-onset ataxia, 22% were tested positive for the biallelic repeat expansion. These patients all presented with neuronopathy, associated to cerebellar ataxia in 80%, with vestibular areflexia in 53%, with cough in 37% and dysautonomia in 23%. The biallelic expansion was found in 62% in case of association of neuronopathy and cerebellar ataxia. The presence of 100% of neuronopathy and quite low frequency of vestibular areflexia can be due to a biais in cases recrutement and/or lack of systematic eye movement recording [23].

Nevertheless, regarding the very high sensitivity of this RFC1 repeat expansion in full familial or sporadic CANVAS, the new definition of CANVAS has probably to be based on genetic.

This review aims at describing and reviewing the clinical, electrophysiological, anatomical, genetic aspect of CANVAS in light of the recent discovery of the genetic aetiology, and discusses differential diagnosis and neuropathology.

## Clinical Presentation

Cerebellar ataxia with neuropathy and vestibular areflexia syndrome (CANVAS) is a slowly progressive late-onset ataxic disorder. According to the series of genetically confirmed cases, age of onset (cough excluded) is around 52 [22, 23] and disease duration at time of examination around 11 (± 7) years. In clinical series, only 16% appears kindred, conforming to an autosomal recessive inheritance [17]. In genetic series, 55% are sporadic [23].

The differences between clinically and genetically defined CANVAS syndromes are presented for 2 cohorts in Table 1.

**Table 1:** Clinical and investigational characteristics in CANVAS cohorts, either clinically or genetically defined

	Szmulewicz et al. 2011	Cortese et al. 2020
	(27 patients)	(100 patients)
	*Clinical definition*	*Genetic definition*
Sporadic/familial cases	23/4	55/45
Sex (M/W)	13/14	45/55
Age at onset (*years*)	60 (33–71)	52 (19–76)
Age at diagnosis (*years*)	71	72 (45–95)
First manifestations	Ataxia, dysesthesia, oscillopsia, dizziness	Ataxia, hypoesthesia, dysesthesia, oscillopsia, dysautonomia
Sensitive neuropathy	100%	95% clinical 100% (95/95) electrophysiological
Hypopallesthesia	–	84%
Cerebellar syndrome	100%	73% clinical 79% with MRI
Cerebellar atrophy on MRI	81% (22/27)	64% (57/91)
Vestibular areflexia	100%	55% clinical 91% (48/53) with videonystagmography
Complete triad	100%	46% clinical 63% with investigations
Cough	–	64%
Dysautonomia	–	48% (20/42)
Normal or brisk tendinous reflexes	–	
*Patellar*		75%
*Achilles*		45%

The ataxic syndrome is explained by a combination of cerebellar postural disorder, proprioceptive deficit due to sensory axonal neuropathy and/or vestibular deficit. However, the timing of onset of the different components may vary, and the diagnosis may be challenging at the beginning of the affection. It was reported a delay of around 10 years to reach the characteristic triad [17]. This means that when two components of the triad are found, the patient has to be regularly retested in time either clinically and/or electrophysiologically to search for the last component.

The recent series of genetically confirmed cases found on clinical and investigations data the complete triad in 69% of cases, isolated neuronopathy in 15%, association of cerebellar ataxia and neuronopathy in 16%, association of vestibular areflexia and neuronopathy in 6% [23]. There were no cases of association of vestibular areflexia and cerebellar ataxia without neuronopathy, and no cases of isolated cerebellar ataxia or vestibular areflexia. However, vestibular clinical or electrophysiological evaluation was performed in only 25% of cases, which suggest that vestibular areflexia might me underestimated in this series [5].

The presenting neurological symptoms are mainly postural imbalance (frequently increased in darkness), dizziness and/or falls, but patients may also primarily complain of sensory symptoms or oscillopsia. Oscillopsia can be permanent and mainly due to downbeat nystagmus associated to cerebellar ataxia. But in CANVAS, oscillopsia is mainly reported during head movements, due to impaired vestibulo-ocular reflex and/or visuo-vestibular ocular reflex. With the disease course, patients may develop dysarthria, dysphagia and symptoms of autonomic dysfuntion. The chronic cough can precede the onset of neurological symptoms by three decades [16, 20, 23].

Gait ataxia is not specific and can be explained by all 3 of the cardinal features of CANVAS: cerebellar ataxia, vestibular ataxia, and a sensory ataxia. The positive Romberg test may reflect either vestibular or sensory deficit.

Clinical evidence of cerebellar dysfunction is given by the combination of cerebellar-type dysarthria, appendicular ataxia, truncal ataxia and cerebellar-type ocular motor deficits. The main frequently reported ocular motor cerebellar signs are jerky smooth pursuit, gaze-evoked nystagmus, downbeat nystagmus and saccadic dysmetria [8, 14, 17, 23–25]. Other clinical ocular motor cerebellar signs such as saccadic intrusions or oscillations, periodic alternating nystagmus, central positional nystagmus, skew deviation or esotropia have not been reported so far [26]. Brain MRI also shows cerebellar atrophy which specificity will be detailed below.

Evidence of bilateral vestibular impairment is given by absent or severely reduced vestibulo-ocular reflex. This one can be clinically demonstrated by abnormal dynamic visual acuity [27], abnormal eye stabilization during slow head rotation using funduscopy [28] and/or mainly using head impulse test [29]. Indeed, to compensate for their deficient VOR, patients with bilateral vestibular impairment use overt saccades to redirect their fovea toward the object of interest after head impulse, a catch-up saccade that can be clinically observed. However, the development of covert saccades occurring during the ongoing head movement may negative clinical HIT and justify systematic ocular motor recording procedures [30].

Compound deficit of vestibular and cerebellar ocular motor stabilizing functions leads to a quite specific deficit of visuo-vestibulo-ocular reflex (VVOR), which is the original feature first described in this syndrome [5, 7, 8, 31]. In case of isolated vestibular or cerebellar deficit, visually guided stabilizing eye movements, respectively vestibulo-ocular reflex and smooth pursuit (and optokinetic reflex), compensate each other to maintain gaze during head movement at low frequency. This is demonstrated by turning a patient’s head from side to side in the yaw plane at about 0.5 Hz while the patient stares at an earth-fixed target (e.g. the clinicians nose) and observing the smooth compensatory eye movements (visuo-vestibulo-ocular reflex, VVOR) [19]. This is not the case anymore when both systems are deficient, leading to clinically detectable catch-up saccades during VVOR (video 1). Video recording of eye movements can also help to identify this sign.

Evidence of sensory neuropathy is given by non-length-dependent multimodality sensory deficit including impairment of vibration sensation, joint position sense abnormalities, impairment of pinprick perception, limb ataxia (heel-knee and finger-nose with eyes closed) and less frequently deficit of pain/temperature [21]. Neuropathic pains are frequent [23]. Tendon reflex may be abolished, mainly Achille’s, but in more than half of the patients, tendon reflexes are preserved or even brisk (including Achille’s) [13, 21, 23, 32]. Electroneuromyography is necessary to confirm the axonal form of this sensory neuropathy. Neuropathology studies disclosed ganglionopathy, even for the vestibular system.

Many patients report a long-standing chronic non-productive cough with normal ENT and pulmonary examination [17]. This cough may have preceded the ataxic manifestations for decades [20, 23]. Some of the patients had demonstration of gastro-oesophageal reflux disease. The mechanism of this cough remains unsettle and it could be vagal neuronopathy and denervation hypersensitivity in the upper airway pathways and oesophagus [20, 23].

Clinical manifestations of dysautonomia, mainly orthostatic hypotension seem to be frequent [17], and more focused studies found very frequent other manifestations of dysautonomia such as cold feet, light-headedness, constipation, dry mouth or eyes, urinary and erectile dysfunction [18, 23]. This is consistent with histopathological evidence of sweat gland denervation [33]. Autonomic dysfunction in CANVAS is supposed to be part of the primary ganglionopathy [18].

Disease progression is slow and 55% may need a walk stick after a mean of 10 years, and 25% a wheelchair after a mean of 15 years of disease duration [23].

## Electrophysiological and Anatomical Aspects

Different investigations are necessary, such as electroneuromyography to confirm or detect signs of neuronopathy, brain MRI to look for cerebellar atrophy and ocular motor vestibular testing to diagnose vestibular areflexia, visuo-vestibulo-ocular reflex deficit and search for cerebellar-induced ocular motor dysfunction.

### Electroneuromyography

Initially, there was no mention of neuropathy in the first description of cerebellar ataxia and bilateral vestibulopathy (CABV) [5], but it was already noticed in a cohort of 53 patients with bilateral vestibulopathy [7] that 7 of them showed cerebellar degeneration and, among them, 4 had a clinical sensory neuropathy. Moreover, in Migliaccio’s article princeps [8], 3 of the 4 CABV have an electrophysiological confirmed sensory neuropathy. We had to wait until 2011 for the denomination CANVAS with the description of 18 patients CABV with an axonal sensitive neuropathy [13], and until 2014 and pathological results to confirm the ganglionopathy [16]. Finally, the first genetically identified cohort [22] confirms the core feature value of neuronopathy since a proportion of 100% sensitive neuropathy is found in both sporadic and familial cases, in contrast to cerebellar syndrome and vestibulopathy.

Electrophysiologically, a ganglionopathy results in a sensory axonal neuropathy without length-dependent pattern, a reduction or even abolition of the H reflexes but relative sparing of motor conduction and F-waves latencies. We propose a literature review of the published cohorts with electrophysiological studies in Table 2 describing qualitatively the data for each parameter (normal, reduced or prolonged, absent) due to a high degree of heterogeneity in the authors’ presentations. In all the studies presented, patients were diagnosed CABV (with Migliaccio’s criteria) or CANVAS (with Szmulewicz’s criteria) depending on the publication study date [8, 13]. The results are consistent with neuronopathy definition showing severe impairment of sensory conduction, with absent or very reduced sensory nerve action potentials (SNAPs) in both lower and upper limbs, albeit with some alteration of motor conduction (in 20%, always slight) congruent with the genetically identified Cortese et al.’s cohort in which motor conduction was normal in 84% of sporadic cases [23]. The moderate decrease in motor action potentials can be explained by a certain degree of axonal degeneration, in proportion to which motor conduction velocities and distal F-wave latencies can be discreetly slowed down or lengthened respectively (in the same proportion, around 20%). Interestingly, we can notice retained H-reflexe despite severe neuronopathy which is congruent with clinical descriptions, although found electrically to a lesser extent (75% of CANVAS cases have clinically retained reflexes in the cohort of Cortese et al. [23]). This remains compatible with the hypothesis of relatively selective impairment of small sensory fibres (sparing Ia fibres) as evidenced by pathological cutaneous silent period [34]*.*

**Table 2:** Neurophysiological results in CANVAS cohorts

	Smulewicz et al. 2011 *n* = 18	Wu et al. 2014 *n* = 26	Smulewicz et al. 2015 *n* = 14	Cazzato et al. 2015 *n* = 4	Burke et al. 2018 *n* = 5	Infante et al. 2018 *n* = 4	Total
Upper limb snaps	15/18 absent 3/18 NR	21/26 absent 4/26 reduced 1/26 normal	14/14 absent	4/4 reduced	3/5 absent 2/5 reduced	4/4 absent	Absent: 57/68 (84%) Reduced: 10/68 (15%) Normal: 1/68 (< 1%)
Lower limb snaps	15/18 absent 3/18 NR	24/26 absent 2/26 reduced	14/14 absent	3/4 absent 1/4 reduced	4/5 absent 1/5 reduced	3/4 absent 1/4 reduced	Absent: 63/68 (93%) Reduced: 5/68 (7%)
CMAP	12/18 normal 6/18 reduced	19/26 normal 7/26 reduced	14/14 normal	4/4 normal	4/5 normal 1/5 reduced	4/4 normal	Normal: 57/71 (80%) Reduced: 14/71 (20%)
DML	12/18 normal 6/18 mildly prolonged	–	10/14 normal 4/14 mildly prolonged	–	–	4/4 normal	Normal: 26/36 (72%) Mildly prolonged: 10/36 (28%)
Motor NCV	12/18 normal 6/18 mildly slowed	–	14/14 normal	4/4 normal	–	4/4 normal	Normal: 34/40 (85%) Mildly slowed: 6/40 (15%)
F-wave latency	9/18 normal 5/18 mildly prolonged 4/18 NR	–	–	–	5/5 normal	4/4 normal	Normal: 18/23 (78%) Mildly prolonged: 5/23 (22%)
Tibial H-reflex	–	–	11/14 absent 3/14 retained	–	–	–	Retained: 3/14 (21%)
Blink reflex	–	–	12/14 abnormal 2/14 normal	–	–	–	Abnormal: 12/14 (86%)
CSP	–	–	7/14 abnormal 7/14 normal	–	–	–	Abnormal: 7/14 (50%)

*SNAPs* sensory nerve action potential, *CMAP* compound motor action potential, *DML* distal motor latency, *NCV* nerve conduction velocity, *CSP* cutaneous silent period, *NR* not recorded

Diagnostic criteria of sensory neuronopathy have been proposed by Camdessanche et al. [35] and are shown in Table 3 (in Annexe). These criteria must be useful in acquired ganglionopathies but less suitable for inherited aetiologies in particular because of the often-bilateral nature of the sensory impairment in these cases, making the item “asymmetrical distribution of sensory loss” maladjusted. Indeed, in genetic neuronopathies, one of the most important neurophysiological elements is electro-clinical discordance between the severity of sensory conduction impairment and the relative paucisymptomatic patients’ condition.

### Cerebellar Atrophy on MRI

**Fig. 1:**
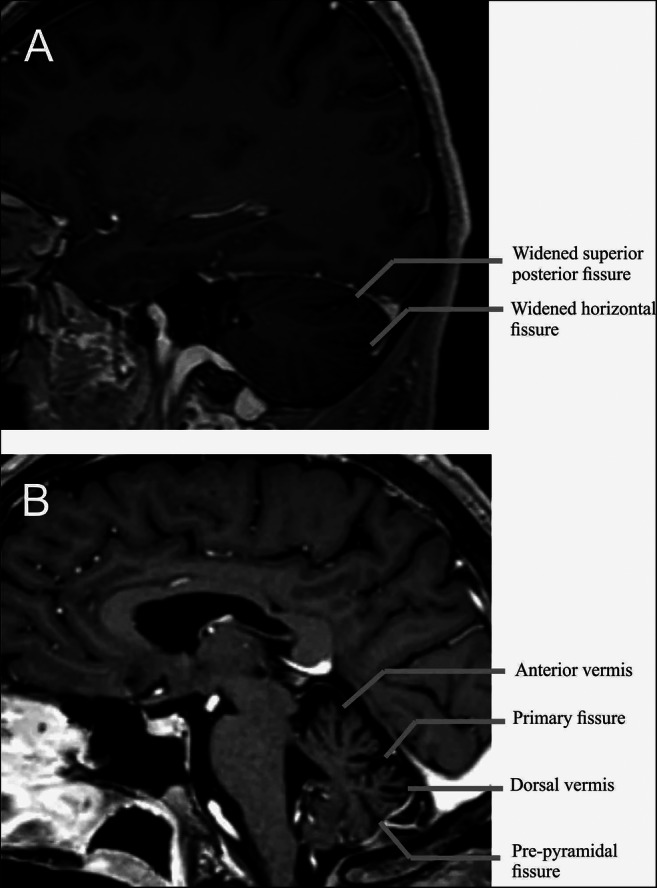
Exemple of typical brain MRI in a patient with CANVAS. (**A**) 3D T1-weighted with gadolinium MRI brain parasagittal section of a 46-year-old CANVAS. This view shows the hemispheric atrophy’s pattern with widening of the superior posterior and of the horizontal fissures, delimiting Crus I. (**B**) 3D T1-weighted with gadolinium MRI brain midsagittal section of a 70-year-old CANVAS. Note the predominant atrophy of the anterior and dorsal vermis and particularly in the part of the dorsal vermis between the primary fissure and the pre-pyramidal fissure corresponding to the vermal lobules VI, VIIa and VIIb

The usefulness of cerebral MRI is closely correlated with cerebellar syndrome since it represents one of the most important investigations in the aetiological assessment of this type of disorder. Besides eliminating many differential diagnoses, a relatively reproducible pattern of cerebellar atrophy has been described [14, 17]. This pattern consists in anterior and dorsal vermial involvement corresponding to lobules VI, VIIa and VIIB, associated with predominant hemispheric atrophy in the posterosuperior and horizontal fissures, delimiting Crus 1 (corresponding to the hemispheric extension of the vermial lobule VII) which, interestingly, is functionally congruent with a part of the oculomotor cerebellum [36] (Fig. 1). This pattern of atrophy is also correlated with the anatomopathological data [15, 16]. In contrast, no specific supra-tentorial abnormality was described. Even if this radiological CANVAS pattern’specificity has not been studied, it does not seem to be shared with seven other neurodegenerative pathologies [37] or with Friedreich ataxia, one of the main differential diagnosis [38], for which cervical medullary atrophy is strongly suggestive.

If the clinical cerebellar syndrome appears to occur later than the peripheral symptomatology (present in 73% of cases in the Cortese cohort, 2020) [23], the MRI anomalies seem to be complementary in the diagnostic process since they were present in 6 patients of this cohort without clinical cerebellar element, justifying the early realisation of this investigation, even without a clinical central involvement.

Finally, MRI spinal cord abnormalities (related to posterior cord injury) have been described later by Cortese et al. [23], reporting on 42 patients genetically determined 45% with posterior cord atrophy and 12% with T2 hypersignals.

### Vestibular and Oculomotor Recording

Objective testing in CANVAS patients can help to confirm bilateral vestibulopathy and quantify the severity and range of the impairment. Moreover, oculomotor testing can allow for early detection of cerebellar impairment in paucisymptomatic patients. Finally, objective measures can help to showcase impairement of the VVOR.

#### Vestibular Impairement

In 2017, a consensus statement regarding diagnostic criteria for bilateral vestibulopathy (BVP) has been published by the Bárány Society in order to help clinicians and improve comparability with regard to scientific research [39]. These criteria associate clinical as well as objective measurements. Patients suffering from BVP are asymptomatic when sitting or lying down, but complain of unsteadiness when walking or standing, movement-induced blurred vision or oscillopsia, and/or worsening of unsteadiness in darkness and/or uneven ground. To confirm the diagnosis, at least one objective measurement (i.e. vestibular testing) has to display an impairment of the vestibular system. Tools used in this regard focus on quantification of the response of the vestibulo-ocular reflex (VOR) after stimulation of the horizontal semi-circular canal. These tools are usually the video head impulse test (vHIT), rotatory chair testing and caloric testing stimulating the vestibular system respectively at medium, low and very-low frequencies. In most CANVAS patients, response to caloric testing is absent or severely reduced bilaterally and vHIT demonstrates gains bellow normative data [14].

**Fig. 2:**
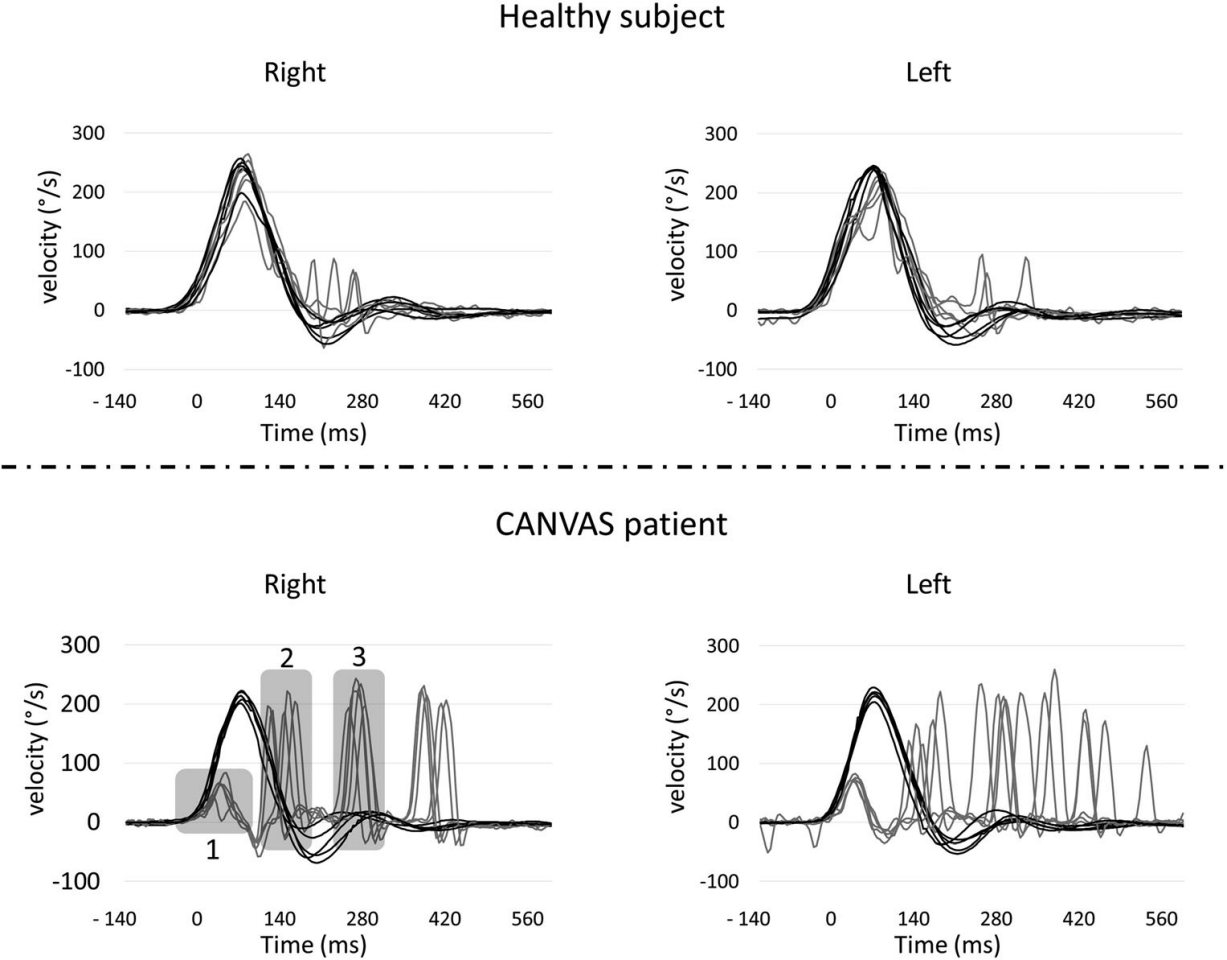
Example of vHIT recording in both a normal subject and a CANVAS patient. vHIT recorded horizontal eye (grey line) and head (dark line) velocities during multiple horizontal head impulses to the right and the left. Top panels represent the responses in a normal subject and bottom panels in a CANVAS patient. For purpose of better comparison, right and left for both eye and head movements have been presented in one unique direction. In the bottom left panel, for the CANVAS patient, the residual VOR (1) does not allow stabilizing gaze during head movement. The patient trigger saccades eigher during head movement (2: covert saccade) and after eye movements (3: overt saccade)

Video head impulse test (or search-coil technique) allows for quantification of the VOR gain during HIT [29] by means of high-speed recording of eye and head movements [40]. Eye movements can be recorded by video and in some teams by scleral-coil technique. In healthy subjects, VOR should suffice to maintain the gaze on the target. In case of vestibular impairment, deficient VOR has to be compensated by catch-up saccades. In addition to VOR gain measurement, vHIT allows for identification of catch-up saccades occurring during the head movements (covert saccades) which are usually not perceived during bedside examination (in contrast to overt saccades occurring after the head movement) (Fig. 2). An impaired bilateral VOR gain of HIT is defined as bilaterally horizontal angular VOR gain < 0.6 on at least 10 impulses. Video HIT is the only tool that can also be used for the evaluation of vertical semi-circular canals [41]. Nevertheless, a disease-specific sparing of the anterior semi-circular canals has been highlighted in BVP rendering gain of the vertical canals a less reliable criterion [42, 43].

During rotatory chair testing, videonystagmography allows for quantification of the VOR in response to a stimulation of both horizontal semi-circular canals at low to middle frequencies. It is thus less sensitive for detection of a unilateral deficit. In BVH, however, VOR gain during rotatory chair testing is greatly reduced as none of the sides can compensate for the deficit of the other side. An impaired VOR gain of the horizontal canals is defined by horizontal angular VOR gain < 0.1 upon sinusoidal stimulation on a rotatory chair (0.1 Hz, Vmax = 50°/s). In addition to VOR gain measurement, rotatory chair testing can also be used for quantification of the visio-vestibulo-ocular reflex (VVOR), cervico-ocular reflex (COR) and visually suppressed vestibulo-ocular reflex (VORS).

Caloric testing has been first described in 1907 but is still used nowadays [44]. The response of the horizontal semi-circular canal to a low/very-low frequency stimulus is evaluated by measuring the caloric induced nystagmus at the culmination phase. Stimulus is achieved by irrigating the outer ear canal for 30 s alternatively with cold (30 °C) and warm (44 °C) water. Caloric testing allows for independent testing of both horizontal semi-circular canals. Bilateral impairment of caloric induced vestibular response is defined by a sum of bithermal maximum peak slow phase velocity on each side <6°/s.

As stated above, diagnostic criteria for BVH do not take into account otolith function. Nevertheless, it can be evaluated with cervical and ocular vestibular evoked myogenic potential (cVEMPs and oVEMPs respectively testing the saccule and the utricule). In CANVAS, similarly to BVH, studies suggest a great variability in responses for VEMP testing [45–47]. Subtypes are still to be defined.

#### Cerebellar-Induced Ocular Motor Impairement

**Fig. 3:**
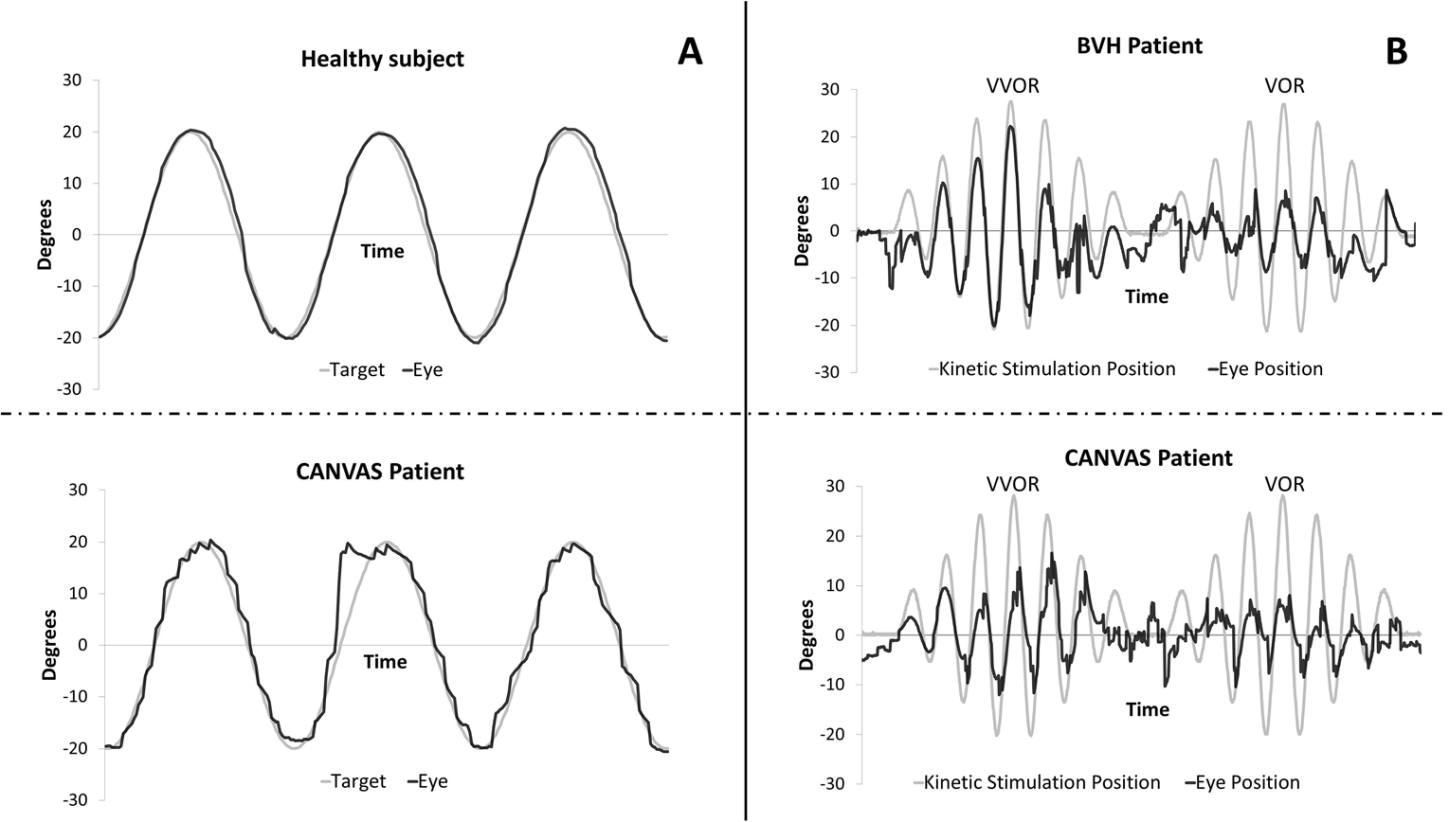
Exemples of eye movement recording during smooth pursuit (**A**), rotatory chair induced vestibulo-ocular reflex/visuo-vestibulo-ocular reflex (VOR/VVOR) (**B**) stimulations. In (**A**), eye position (dark line) and displacement of the triggering visual target (grey line) are plotted in a health subject (top) and a CANVAS patient (bottom). CANVAS patient shows jerky smooth pursuit. In (**B**) , eye position (dark line) and kinetic chair position (grey line) are plotted in a patient with isolated bilateral vestibular hypofunction (BHV) (top) and a patient with CANVAS (bottom). For purpose of better comparison, both eye and chair movements have been presented in one unique direction. In both patients VOR is deficient, but only the CANVAS patient shows the quite specific VVOR deficit

Videonystagmography can help to identify ocular motor signs of cerebellar impairment. Saccadic testing in CANVAS patients often shows dysmetric saccades with a normal velocity [8, 19, 48]. Smooth pursuit is typically impaired showing square waves, broken pursuit, and decreased gain during both horizontal (Fig. 3a) and vertical target movement [8, 14]. Opposite to what is usually found in patients with cerebellar impairment of slow eye movements, visually suppressed VOR (VSVOR) in “falsely normal” in CANVAS patients (< 0.1), due to absence of VOR [8, 14].

Cervico-ocular reflex (COR) is an ocular stabilization reflex that is elicited by rotation of the neck. It usually contributes little to gaze stability in normal subjects [49] but is greatly enhanced in patients with BVH to compensate for VOR deficit [50]. It has been suggested that this compensatory enhancement of the cervico-ocular reflex is absent in patients with CANVAS [5, 6].

#### Visuo-Vestibular Ocular Reflex

As explained earlier, patients suffering from CANVAS have an impairment of all networks of slow stabilizing eye movements (vestibulo-ocular reflex, optokinetic reflex and smooth pursuit) resulting in a deficient visuo-vestibular ocular reflex (VVOR). Evaluation of VVOR can be done by using videonystagmography under rotational chair testing or a technique of portable high-speed videooculography equipment [19] (Fig. 3b). Thresholds for normal VVOR gain depend on normative data relative to the tools used although normal gain is uselly close to 1 and not under 0.95 [51]. VVOR gains in CANVAS patients have been reported around 0.63 or 0.4 depending on the series [51, 52]. It is likely that VVOR gain gradually decreases as neural degeneration progresses.

## Genetic Diagnosis

The topicality of this syndrome is marked by the discovery of the genetic responsible abnormality of biallelic intronic AAGGG repeat expansion in the replication factor C subunit 1 (RFC1) [22].

Assuming the transmission was autosomal recessive, the study’s methodology consisted in combined use of classical genetic investigations and whole-genome sequencing (WGS). Indeed, the first step consisted in linkage analysis based on comparison between siblings affected or not haplotypes in order to reduce the genomic search zone to a 1.7 Mb region. Whereas whole-exome sequencing (WES) was inconclusive, whole-genome sequencing (WGS) was performed in this region and identified, by visual inspection of the aligned read pairs, a reduced read depth encompassing a short tandem repeat (STR) with as reference (AAAAG)_11_ replaced by a variable number of (AAGGG) units (detected on both sides of the STR). The pathological repeat (AAGGG) has then been confirmed with repeat-primed PCR (RP-PCR) using specific primers. Standard flanking PCR failed to amplify this region in CANVAS patients suggesting the presence of a large expansion (the elongation time depends on the polymerase used and limits the amplification’s maximum size). This was confirmed by amplification with long range PCR (using a polymerase with a longer elongation time) and Sanger sequencing. The expansion sizes were estimated by southern blot with a mean of 1000 repeats (range 400 to 2000) *and no correlation was found between age at onset and the size of the repeat*. These results have been confirmed 3 months later by another independent study with a similar methodology using bioinfortmatics tools [53].

First, four different repeat conformations have been observed [22]: the wild type (AAAAG)_11_ and 3 longer expansions sequences (AAAAG)_n_, (AAAGG)_n_ and the pathological (AAGGG)_n_. Two other conformations were described later by Akcimen et al. [54], probably corresponding to the 3% of control cases whose exact sequence could not be determined by Cortese et al.: (AAGAG)_n_ and (AGAGG)_n_. Furthermore, it has been suggested that the conformation (AAAGG)_n_/(AAGGG)_n_ could be associated with CANVAS and thus may correspond to a symptomatic compound heterozygote [53].

The interest of this brief methodological description lies in the fact that the genetic tests routinely available today follow these steps and help to understand that a positive case is defined by an absence of possible amplification in standard PCR (short range) of the intron 2 of RFC1 (thus objectivizing a large expansion, contrary to healthy subjects for whom amplification is realisable) with a positive target RP-PCR (AAGGG).

Pentanucleotide expansion (AAGGG) occurs in the 3′ end of an Alu element: AluSx3. Alu elements are repeating elements about 300 bp, with a 3′ end part containing a long A-rich region considered highly polymorphic. Indeed, over the evolution, retrotransposons active elements degrade by poly(A) tail shortening or inactivation by guanine/cytosine interruption [55]. The main hypothesis adopted is that the irruption of guanine/cytosine alters the three-dimensional structure of DNA, making it more vulnerable to external damaging agents, thereby perpetuating genomic instability and thus initiate and promote the expansion of repeats. This is supported by the fact that other pathologies (and often neurological or neurodegenerative disorders) are linked to localized expansion repetitions on Alu elements such as many SCAs and Friedreich’s ataxia [56]. This therefore presupposes the existence of an original genetic event (mutation from AAAAG to AAAGG or AAGGG), and thus a more recent common ancestor (MRCA) who lived approximately 25,000 years ago in Europe [53].

In order to estimate the pathological allelic carrier frequency, Cortese et al. screened more than 300 controls and observed a heterozygous rate about 0.7% and thus an estimated homozygous prevalence of 1/20,000 [22]. Moreover, they evaluated the diagnostic value of this test screening a sporadic late-onset ataxia cohort of 150 patients and found 22% carrying the recessive AAGGG repeat expansion [22]. This percentage increased to 63% in the cases with sensory neuronopathy and cerebellar impairment and to more than 90% in cases with the full CANVAS syndrome. These data suggest that this syndrome could be a frequent cause of late-onset ataxia, probably of the same order of magnitude as the Friedreich’s ataxia [57–59].

## Differential Diagnosis

Progressive ataxia due partial or complete combination of cerebellar ataxia, sensory neuropathy and vestibular deficit, as well as association to dysautonomia and chronic cough, is not specific to CANVAS syndrome. Differential etiologies or syndromes are declined below. Note however that, when present, the vestibular deficit is usually profound in CANVAS, resulting when associated to cerebellar ataxic ocular motor disorders, to the quite specific VVOR deficit. Regarding what was previously suggested as testing in diagnosing CANVAS syndrome [14, 19], the recent discovery and access to genetic test for CANVAS change our diagnostic strategy in looking for this genetic disorder first, then to other possible diseases if negative.

### Known Genetic Disorders

The association of cerebellar ataxia, vestibular deficit and sensory neuropathy may be observed in known genetic disorders such as Friedreich ataxia, SCAs, mainly SCA3 and SCA1 and other phenotypes of mitochondrial diseases.

In adult-onset Friedreich ataxia, cerebellar impairment and sensory neuronopathy can be accompanied by vestibular deficit, but much less profound than that of CANVAS [3, 60]. Indeed, horizontal HIT mean gain was found around 0.50 in the Fahey’s study. The younger age at disease onset and hearing deficit can also help to differentiate it from CANVAS. However, Friedreich ataxia remains one of the most obvious differential diagnosis.

Vestibular areflexia in SCA3 has been reported later on [1, 2], and this disease is also associated to both sensory and motor neuropathy of mixed type with axonal and demyelinating characteristics [61]. In SCA1, both neuropathy and vestibular deficit is observed, but still less profound than in CANVAS [2]. A novel variant of ELF2 gene has been recently observed in a British family with presumed autosomal dominant CANVAS with incomplete penetrance and variable expressivity [48]. In this study, the 3 sibblings presented the complete phenotype of CANVAS, and the pathogenic role of the ELF2 variant was suggested. Further studies are needed to confirm a potential new SCA with CANVAS phenotype. The age of onset and the autosomal dominant inheritance should differentiate SCA from CANVAS, but sporadic cases may be challenging.

Within mitochondrial diseases, association of sensory neuropathy and cerebellar ataxia are prevalent notably in the phenotype of mutations in the nuclear POLG1 gene [62]. While vestibular dysfunction has not been reported in POLG1 phenotype, it seems to be quite frequently observed in mitochondrial disease, most of them associated to sensorineural hearing loss [63]. Therefore, looking for POLG1 mutation could be recommended if CANVAS mutation is not found.

### Multiple System Atrophy

Because of dysautomic symptoms, the cerebellar form of multiple system atrophy (MSA-c) can be suspected, but the absence of vestibular deficit [64, 65], the more rapid disease progression and brainstem atrophy might help to differentiate from CANVAS [18]. Furthermore, RFC1 expansion was not found in such patients [66].

### Unspecific Neuropathy with Vestibular Deficit or with Chronic Cough

The association of neuropathy and vestibular deficit has been observed in numerous etiologies such as Charcot Marie Tooth [67–69], CIDP and other inflammatory neuropathies [11, 70, 71] and more generally in peripheral neuropathy [12]. However, in none of the reported cases, the vestibular deficit was as profound as in CANVAS, which rise the suggestion that even if cerebellar ataxia, dysautonomia and cough can be absent, CANVAS diagnosis has to be based on profound vestibular deficit and axonal sensory neuropathy, without prominent motor neuropathy neither sensorineural hearing loss.

There is a profuse literature on chronic cough resulting from neurogenic causes that could be secondary to local sensory nerve damage caused by inflammatory, infective and allergic factors [72]. The association of chronic cough and neuropathy has been reported in Holmes-Adie syndrome [73], MPZ (peripheral myelin protein zero) mutation associated neuropathy [74] and autosomic dominant sensitive neuropathy with gastro-oesophageal reflux [75]. In none of these etiologies cerebellar syndrome or vestibular areflexia was observed. Chronic cough is therefore an associated symptom that may help to orient the diagnosis if associated to at least two out of the main triad: cerebellar ataxia, neuronopathy or vestibular areflexia.

### Alcoholic Intoxication

The association of peripheral neuropathy and cerebellar ataxia can be observed in chronic alcoholic intoxication, and vestibular deficit has been observed in Wernicke’s encephalopathy [76, 77], but the time course of these manifestations does not correspond to the very slowly progression of different symptoms in CANVAS.

## Neuropathology

Neuropathology studies over these last 10 years help to better understand CANVAS and to confirm that axonal sensory neuropathy, vestibular areflexia and dysautonomia were related to a ganglionopathy.

Nerve biopsy demonstrates severe axonal neuropathy [8], predominantly in myelinated fibres [22], with non significant regeneration and no active axonal degeneration, no demyelination [23]. Axonal neuronal loss was found to be associated to severe neuronal loss of the sensory nerve ganglia, of the vestibular, facial and trigeminal nerves [15, 16], and of the dorsal root of spinal cord [16]. The spinal cord neuropatholgy also showed severe atrophy of dorsal root and posterior column [16]. At cerebellar level, Pukinje cell loss predominates with some preservation of deep cerebellar nuclei and atrophy predominates at anterior and superior level and VIIa vermal part (as demonstrated on MRI) [16]. In these different cases, preservation of acoustic nerve and its spiral ganglia, peripheral vestibular apparatus, anterior and lateral horns and lateral thoracic columns of the spinal cord were observed. 

## Physiopathology

According to Hannan et al. [78], the main pathophysiological mechanisms of non-coding tandem repeat are represented by toxic gain of RNA function (including formation of RNA foci and repeat-associated-non ATG translation, i.e. translation of a peptide from a tandem repeat without ATG start codon leading to toxic effect of the resultant peptide), toxic gain of protein function and loss of gene expression or function. Although the recessive mode of transmission is more in favour of a loss of gene function as in Friedreich’s disease (also underpinned by intronic repeat expansion), this could not be demonstrated yet. Indeed, Cortese et al. [22] did not find any qualitative (via RNA sequencing, RNA-seq) or quantitative (via quantitative reverse transcription PCR) alteration of RFC1 or its satellite genes. Moreover, the anatomopathological study did not find any endogenous RNA foci and their comparative study of RFC1 protein level between healthy controls and CANVAS patients, with western blot analysis, both in peripheral tissues (fibroblasts and lymphoblasts) and in the brain, did not find any significant difference.

RFC1 codes for a subunit of replication factor C (RFC), which has a crucial role in DNA replication and repair [79]. Interestingly, other pathologies are related to proteins involved in DNA repair, foremost among which are ataxia with oculomotor apraxia types 1 and 2 and particularly ataxia telangiectasia [80], presenting as common characteristics with CANVAS a progressive cerebellar involvement associated with neuropathy, suggesting a particular susceptibility of these 2 tissues to DNA repair failure.

In summary, although still very uncertain and unproven, the main pathophysiological hypothesis suggests a mutation in a polymorphic zone, favouring during evolution the expansion of repeats modifying the expression of a gene coding for a protein involved in DNA repair, leading to damage to the most vulnerable tissues, notably the cerebellum and peripheral nerves, in particular the small fibres (Aδ and C). This differential fibre nerve damage may also explain the chronic cough. Physiologically, the cough corresponds to a reflex arc triggered by stimulation of polymodal receptors located mainly in the larynx and in the tracheobronchial tree. These nociceptors correspond to the myelinated Aδ (rather excitatory) and non-myelinated C (which may be excitatory or inhibitory) fibres which transmit information to the still poorly known centres of the brain stem, including the solitary nucleus. The efferent pathways are coming largely from the ambiguous nucleus and finally stimulate the muscles that cause the cough [81]. In that respect, the relatively specific involvement of the C fibres may lead to a decrease in their inhibitory effect resulting in hyperexcitability of the efferent neurons of the solitary nucleus.

## Conclusion

CANVAS is a nice story of a new disease which discovery involved the combined competences of specialists in neurootology, movement disorders/ataxia, peripheral nerve/neuromuscular diseases, neuropathology and finally neurogenetic. The last genetic discovery of biallelic RFC1 expansion explains most of the familial and sporadic cases. When the triad is complete, the sensitivity of the RFC1 expansion is very high, even in the sporadic cases. On the other hand, the core manifestation of the disease is the neuronopathy, and either cerebellar ataxia and/or vestibular areflexia may be lacking. Systematic clinical and electrophysiological search of eye movement disorders still lacks in the more recent cohort. And finally some of full phenotype of CANVAS may be explained by other diseases such as the novel variant of ELF2 gene or not explained yet. Therefore, one remaining question is whether the term CANVAS should be reserved to the clinically defined syndrome and/or the genetically (RFC1 expansion) defined disease.

### Electronic Supplementary Material

ESM 1 (MP4 7161 kb)

## Annexe. Neuronopathy Diagnostic Criteria Proposed by Camdessanche et al. [35]

**Table 3:** A. In a patient with a clinically pure sensory neuropathy a diagnosis of sensory neuronopathy is considered as possible if score > 6.5

Ataxia in the lower or upper limbs at onset or full development	+ 3.1
Asymmetrical distribution of sensory loss at onset or full development	+ 1.7
Sensory loss not restricted to the lower limbs as full development	+ 2.0
At least 1 SAP absent or 3 SAP < 30% of the lower limit of normal in the upper limbs, not explained by entrapment neuropathy	+ 2.8
Less than two nerves with abnormal motor nerve conduction studies in the lower limbs	+ 3.1
If > 6.5, a diagnosis of SNN is possible	Total:
B. A diagnosis of sensory neuronopathy is probable if the patient’s score is > 6.5 and if	
1. The initial workup does not show biological perturbations or ENMG findings excluding SNN and	
2. The patient has one of the following disorders: onconeural antibodies or a cancer within 5 years (Graus et al., 2004), cisplatin treatment, Sjögren’s syndrome (Vitali et al., 2002)	
3. Or MRI shows high signal in the posterior column of the spinal cord	

*SNN* sensory neuronopathy
